# Nanocomposite Polymer Gel Electrolyte Based on TiO_2_ Nanoparticles for Lithium Batteries

**DOI:** 10.3390/membranes13090776

**Published:** 2023-09-01

**Authors:** Nikita A. Slesarenko, Alexander V. Chernyak, Kyunsylu G. Khatmullina, Guzaliya R. Baymuratova, Alena V. Yudina, Galiya Z. Tulibaeva, Alexander F. Shestakov, Vitaly I. Volkov, Olga V. Yarmolenko

**Affiliations:** 1 Federal Research Center of Problems of Chemical Physics and Medicinal Chemistry RAS, 142432 Chernogolovka, Russia; chernyak@icp.ac.ru (A.V.C.); khatmullinakg@mpei.ru (K.G.K.); guzalia@icp.ac.ru (G.R.B.); gvinok@icp.ac.ru (A.V.Y.); galia@icp.ac.ru (G.Z.T.); a.s@icp.ac.ru (A.F.S.); vitwolf@icp.ac.ru (V.I.V.); oyarm@icp.ac.ru (O.V.Y.); 2 Scientific Center in Chernogolovka of the Osipyan Institute of Solid State Physics RAS, 142432 Chernogolovka, Russia; 3 Department of Chemistry and Electrochemical Energy, Institute of Energy Efficiency and Hydrogen Technologies (IEEHT), National Research University “Moscow Power Engineering Institute”, 111250 Moscow, Russia; 4 Faculty of Fundamental Physical and Chemical Engineering, M. V. Lomonosov Moscow State University, 119991 Moscow, Russia

**Keywords:** nanocomposite polymer gel electrolytes, Li//LiFePO_4_ battery, TiO_2_ nanoparticles, PFG NMR, self-diffusion coefficients, ionic conductivity

## Abstract

In this article, the specific features of competitive ionic and molecular transport in nanocomposite systems based on network membranes synthesized by radical polymerization of polyethylene glycol diacrylate in the presence of LiBF_4_, 1-ethyl-3-methylimidazolium tetrafluoroborate, ethylene carbonate (EC), and TiO_2_ nanopowder (d~21 nm) were studied for ^1^H, ^7^Li, ^11^B, ^13^C, and ^19^F nuclei using NMR. The membranes obtained were studied through electrochemical impedance, IR-Fourier spectroscopy, DSC, and TGA. The ionic conductivity of the membranes was up to 4.8 m Scm^−1^ at room temperature. The operating temperature range was from −40 to 100 °C. Two types of molecular and ionic transport (fast and slow) have been detected by pulsed field gradient NMR. From quantum chemical modeling, it follows that the difficulty of lithium transport is due to the strong chemisorption of BF_4_^–^ anions with counterions on the surface of TiO_2_ nanoparticles. The theoretical conclusion about the need to increase the proportion of EC in order to reduce the influence of this effect was confirmed by an experimental study of a system with 4 moles of EC. It has been shown that this approach leads to an increase in lithium conductivity in an ionic liquid medium, which is important for the development of thermostable nanocomposite electrolytes for Li//LiFePO_4_ batteries with a base of lithium salts and aprotonic imidasolium ionic liquid.

## 1. Introduction

TiO_2_ nanoparticles are of interest in many applications [1]. They can be used for catalysis and photocatalysis, as an effective UV filter and pigment for paints, in pharmaceuticals, and as components of electrolytes and electrodes in power sources. TiO_2_ exists as several crystalline modifications [1]. Anatase and rutile (tetragonal singony) and brukite (rhombic singony) are found in nature. Anatase and rutile differ markedly in terms of density (3.78 and 4.24 g·cm^−3^, respectively) and dielectric constant (ε). Anatase has a low ε = 48, and rutile has a very high ε = 130. Since both anatase and brukite are irreversibly transformed into rutile upon heating, it is possible to obtain TiO_2_ nanoparticles consisting of a mixture of several phases [2], and there are approaches that allow for control over obtaining phases of anatase or rutile [3]. It is possible to synthesize TiO_2_ in the process of obtaining nanocomposites with polymer networks [4].

Initially, nanoparticles were used in electrochemical devices to improve the conductivity of solid polymer electrolytes by reducing their degree of crystallinity. Later, it was found that the introduction of nanoparticles not only improves conductivity but also enhances the mechanical properties of the films and reduces the resistance at the electrodes surface.

Nanocomposite gel electrolytes, which contain liquid aprotonic solvents, have enhanced conductivity. The use of non-flammable ionic liquids (ILs) as plasticisers instead of unsafe organic solvents is attractive. ILs have unique physicochemical properties: thermal stability, insignificant volatility, low combustibility, and high ionic conductivity [5,6,7,8,9]. Owing to these properties, they have found use as components of electrolytes for various electrochemical devices, supercapacitors, and lithium–ion batteries [10,11,12,13,14].

When polymer matrices are plasticised with ionic liquids, their thermal and mechanical properties are also improved [15,16]. However, when introducing ILs into lithium-conducting polymer electrolytes, the competitive transport of different ions occurs, and very low transport numbers for Li^+^ cations are realised [13,14,16]. It is possible to increase the transfer numbers of target ions and to improve the electrochemical properties of the gel system by introducing ceramic nanofillers (for example, Al_2_O_3_, SiO_2_, ZrO_2_, TiO_2_, SnO_2_, etc.) into gel polymer electrolytes [17,18,19,20,21,22,23]. These electrolytes were named nanocomposite polymer gel electrolytes (NPE). They may be prepared by casting from a polymer solution or by synthesis in situ from the oligomer.

NPEs produced by casting from solution [15,16,24,25] retain a porous structure due to the dispersion of nanofiller. This leads to an increase in the absorption capacity of the plasticiser, which reduces leakage and reactivity and improves safety.

Through the radical polymerisation of oligomer in ionic liquid medium with the addition of ethylene carbonate, polymer electrolyte films are obtained in one stage [26]. It is such electrolyte samples that are convenient to study by NMR in order to reveal the mechanism of ionic and molecular transport in these complex systems [27], since polymer electrolyte samples can be obtained directly in NMR ampoules. Therefore, this approach is convenient to use for the synthesis of nanocomposite polymer electrolytes in order to study the features of the ion transport mechanism in this complex system, especially since the conductivity on lithium cations is expected to increase upon the addition of TiO_2_ nanoparticles.

In the present work, an electrolyte system based on polyethylene glycol diacrylate, LiBF_4_ salt, tetrafluoroborate 1-ethyl-3-methylimidazolium ionic liquid with ethylene carbonate additives and introduction of TiO_2_ nanoparticles in the amount of 2 and 6 wt.% was investigated. High-resolution NMR and pulsed field gradient NMR using ^1^H, ^7^Li and ^19^F nuclei methods were used in combination with electrochemical impedance spectroscopy and quantum chemical modelling to study the mechanism of ionic and molecular transport.

The studies on ^7^Li and ^19^F make it possible to monitor the ion mobility of the lithium cation and BF_4_^−^ anion, respectively. The study on ^1^H provides information about the mobility of the 1-ethyl-3-methylimidazolium cation and ethylene carbonate molecules.

## 2. Materials and Methods

### 2.1. Materials

TiO_2_ nanoparticles (average particle size 21 nm, Aldrich, Evonik Resource Efficiency GmbH, Hanau-Wolfgang, Germany, purity ≥ 99.5%) were used to fill the electrolyte polymer matrix. AEROXIDE^®^ TiO_2_ P 25 is a fine particulate, with a high specific surface area 35–65 m^2^ g^−1^ (BET), mp 1850 °C, density 4.26 g mL^−1^ at 25 °C. TiO_2_ P 25 has a unique combination of anatase and rutile crystal structure. The SEM image of the initial TiO_2_ powder is shown in Figure S1a, ESI.

**Figure 1:**
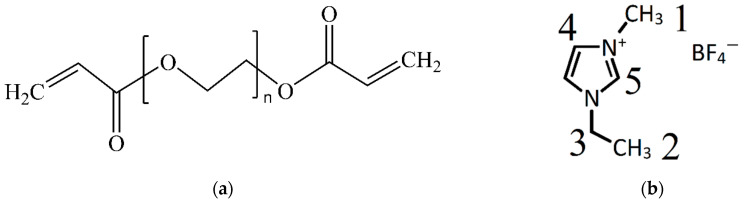
Structures of (**a**) polyethylene glycol diacrylate (PEGDA) and (**b**) ionic liquid EMIBF_4_, where digits indicate the sites of the 1H and 13C atoms (for the description of the NMR spectra).

Polyethylene glycol diacrylate (PEGDA, Aldrich, M_n_ = 700, *T*_melt_ = 12–17 °C) was used to obtain a three-dimensional network matrix for the polymer electrolyte. The structure of PEGDA is shown in Figure 1a.

LiBF_4_ (purity 98%) was used as electrolyte salt, ethylene carbonate (EC, Aldrich, *T*_melt_ = 36 °C, purity ≥ 99%) was used as electrolyte solvent, and 1-ethyl-3-methylimidazolium tetrafluoroborate (EMIBF_4_, Aldrich, purity ≥ 98%) was used as ionic liquid. The structure of EMIBF_4_ is shown in Figure 1b.

All chemical reagents and diluents were acquired from Sigma–Aldrich and were used as received.

The radical polymerization initiator, benzoyl peroxide (PB, Aldrich), stored in water (30%) was recrystallized from chloroform, followed by drying at 20 °C in air and then in a vacuum.

### 2.2. Synthesis of Nanocomposite Polymer Electrolyte

The nanocomposite polymer electrolyte was synthesized by the radical polymerization of PEGDA in the presence of the radical initiator PB. Poly(ethylene glycol) diacrylate, which contains ethylene oxide units, forms a three-dimensional network during radical polymerization of PEGDA. The double bonds C=C at the terminal groups of PEGDA open when attacked by a radical initiator during heating. A three-dimensional polymer network of this structure can hold the liquid aprotic solvents and the ionic liquids.

The composition of the polymerizable mixture was as follows: PEGDA, LiBF_4_, EC, EMIBF_4_, TiO_2_, and 1 wt.% PB. After mixing the initial components, the solution was poured into a germetic flat glass reactor with a Teflon spacer with a thickness of 0.2–0.3 mm. The mixing of the components and pouring of the solution of the polymerizable composition into a glass reactor were both carried out inside the MBraun argon glove box.

The mixture was cured in a laboratory oven according to the following regime: 60 °C for 3 h, 70 °C for 1 h, and 80 °C for 1 h [28]. The synthesis procedure produced white films with 0.2–0.3 mm thicknesses, as determined by the thickness of the Teflon spacer. Optical photographs of the film surface are shown in Figure S1, ESI.

In order to study samples through the NMR method, the NPEs were synthesized in closed glass capillaries with a diameter of d = 4 mm, l = 50 mm. The capsules with NPE were closed and placed in standard 5 mm ampoules for the NMR examination.

The preparation of the electrolyte solution and synthesis in different reactors (films and capillaries) were carried out simultaneously under the same conditions.

### 2.3. Differential Scanning Calorimetry (DSC) Method

The glassy transition temperature of the NPE determined from differential scanning calorimetry (DSC) data obtained using a DSC 822e Mettler–Toledo instrument ((Kutznacht an der Zürichsee, Switzerland)) with the Star software (Website: https://www.mt.com/hk/en/home.html). The temperature interval from −150 to 50 °C, and a scanning rate of 5 deg min^−1^ were applied.

### 2.4. Thermogravimetric Analysis (TGA) Method

The TGA data for the samples were obtained on a TGA/SDTA851 Mettler–Toledo instrument (China) in the temperature range from 20 to 150 °C at a heating rate of 5 deg/min.

### 2.5. Electrochemical Methods

In order to measure the conductivity of NPE film samples using the electrochemical impedance method in symmetrical stainless steel (SS)//SS cells, a Z—2000 impedance meter (Elins, Chernogolovka, Russia) was used in the frequency range from 1 Hz to 600 kHz with a signal amplitude of 10 mV. The cell impedance was detected in the temperature range from −40 to 100 °C. Four measurements were carried out for each sample. The measurement error was not higher than 2%. The parameters of equivalent circuit models were calculated using the ZView2 package.

The electrochemical performance of the Li//LiFePO_4_ (LFP) batteries was evaluated using a BTS—5 V 10 mA battery analyzer (Neware Technology Ltd., Shenzhen, China) by performing charge/discharge cycling at a current density of 17 mAh g^−1^ in a range of 2.6–3.8 V. The electrochemical performance of LFP was evaluated in coin-type CR2032 lithium batteries. The cathode composition consisted of 70 wt.% of LFP, 20 wt.% of conductive carbon black (Timical Super C65) and 10 wt.% of PVDF polymer binder (Kynar Flex HSV 900, Arkema, Colombes, France). NPE was used as a separator. The electrodes were pretreated with liquid electrolyte 1 M LiTFSI DOL/DME (2:1 vol.).

### 2.6. Pulsed Field Gradient NMR

NMR measurements on a Bruker Avance—III 400 MHz NMR spectrometer (Bruker, Rheinstetten, Germany) equipped with the diff60 gradient unit (the maximum field gradient amplitude was 30 Tm^−1^) were carried out at a temperature of 22 ± 1 °C. The ^1^H (diffusion of solvent molecules and ionic liquid IL, IL^−^), ^7^Li (diffusion of lithium cations), and ^19^F (diffusion of anions) NMR measurements were carried out with operating frequencies of 400, 155.5, and 376.5 MHz, respectively. The stimulated spin–echo sequence was applied. The details of self-diffusion coefficient measurements are given in References [29,30]. The experimental NMR parameters of pulse sequences were the following: π/2 pulse was 9 μs (^1^H), 9 μs (^7^Li), and 10 μs (^19^F); gradient pulse duration time δ was 1 (^1^H), 1 (^7^Li), and 3.0 (^19^F) ms; diffusion time was 19.7 (^1^H), 19.7 (^7^Li), and 49.0 (^19^F) ms; repetition time was 3 s; and the diffusion 32 steps with the maximum field gradient amplitude g were 3.5 (^1^H), 11.5 (^7^Li), and 4.0 (^19^F) Tm^−1^. The measurement error of the self-diffusion coefficients was 5%. The temperature dependences of the diffusion coefficients were measured in the temperature range from 22 to 60 °C.

### 2.7. High-Resolution NMR

High-resolution spectra for ^1^H, ^7^Li, ^11^B, ^13^C, and ^19^F were recorded on a Bruker Avance III 500 MHz NMR spectrometer. The measurements at frequencies of 500, 194, 160, 126, and 471 MHz for ^1^H, ^7^Li, ^11^B, ^13^C, and ^19^F, respectively, were carried out at room temperature (22 ± 1 °C). The chemical shift scale was calibrated with the DMSO—d6 signal in the capillary as an external standard (2.50 ppm for ^1^H). The ^1^H, ^7^Li, and ^19^F NMR spectra were obtained using the standard sequence π/2 pulses, FID. No signal accumulation was applied. In order to obtain the ^13^C NMR spectra, a standard sequence from the TopSpin (Bruker, Rheinstetten, Germany) zgpg30 library was used. The sequence is an accumulation of signals from 30° pulses with the suppression of the ^1^H spin–spin interaction for the duration of all the experimental times. The number of repetitions was ns = 512, and the delay between the repetitions sequence was d1 = 1.0 s.

### 2.8. Quantum Chemical Modeling

The structure of complexes of different ions with solvent molecules and TiO_2_ was studied using the nonempirical Perdew–Burke–Erzernhof (PBE) exchange–correlation functional [31] using the extended basis Ti [19s16p11d5f/6s5p3d1f], H [5s1p/2s1p], B, C, N, O, F [10s7p3d/3s2p1d], Li [10s7p3d/4s3p1d]. H [6s2p/2s1p]. The Hirschfeld method [32] was used to calculate atomic charges. The Priroda package [33] was used for all of the calculations carried out at the Joint Supercomputer Center of the Russian Academy of Sciences.

## 3. Results and Discussion

### 3.1. NPEs Synthesis

The compositions of the nanocomposite polymer electrolytes synthesized with the introduction of 2 and 6 wt.% TiO_2_ nanopowder are given in Table 1. The composition of NPE0 without nanoparticles synthesized and studied by us previously [28] is presented for comparison. The nanopowder amounts were chosen as 2 and 6 wt.% because of the preliminary studies of the network polymer electrolytes based on polyethylene glycol diacrylate. It has previously been shown [34] that the highest conductivity is characteristic of the compositions with 2 and 6 wt.% SiO_2_ nanoparticle additives to the gel electrolytes PEGDA, LiBF_4_, and γ—butyrolactone. In addition, the dependence of the conductivity of the solid NPE containing PEGDA, LiBF_4_, and EC on the amount of TiO_2_ nanoparticles (0, 2, 4, 6, and 8 wt.%) without EMIBF_4_ was studied in this work. The exact NPE compositions are given in Table S1, ESI. It was found that the composition with 6 wt.% TiO_2_ had the highest conductivity (Figure S2, ESI). Solid polymer electrolytes usually exhibit a maximum ionic conductivity as a function of the amount of nanopowder introduced, which is caused by the formation of conductive paths involving nanoparticles [35]. Thus, the percolation threshold with the introduction of 6 wt.% TiO_2_ is reached.

### 3.2. DSC of NPEs

**Figure 2:**
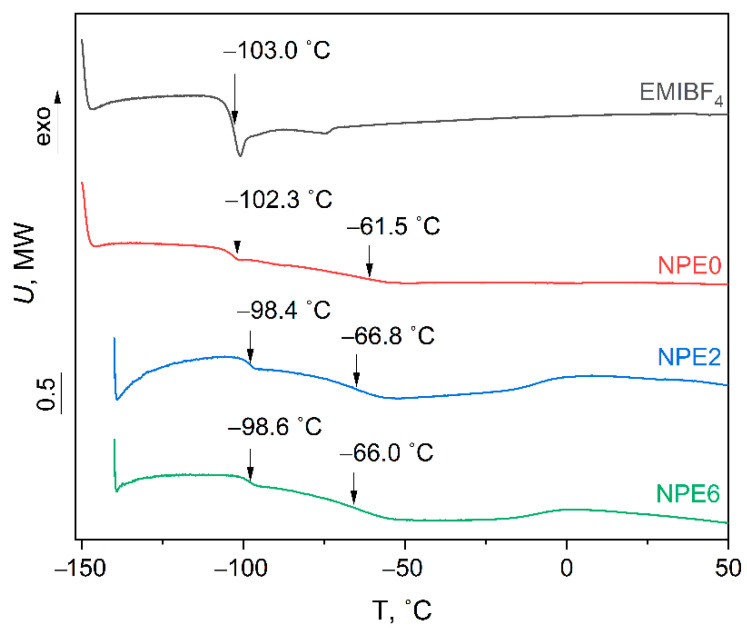
DSC curves for all NPE compositions and ionic liquid EMIBF_4_.

The DSC curves for three polymer electrolyte compositions (Table 1) and initial ionic liquid EMIBF_4_ are presented in Figure 2.

Figure 2 and Table S2, ESI show that only phase glassy transitions are observed in all NPE compositions. The NPE compositions exhibit two glassy transition temperatures.

The first glass transition temperature is assigned to the network polymer matrix based on PEGDA. This transition is very extended (22–33 °C). It indicates a slow glassy transition of the three–dimensional polymer PEGDA matrix (Table S2, ESI).

The second glassy transition temperature is attributed to the phase transition of the ionic liquid (−103 °C) to NPE (according to the DSC data). This transition occurs very rapidly within 3 to 4 degrees (Table S2, ESI).

The earlier studies [28] by isothermal calorimetry showed that the radical three-dimensional polymerization of PEGDA in the ionic liquid occurs for temperatures higher than 60 °C, and the limiting polymerization depths is quickly achieved at 80 °C. The established fact of decreasing the cross-linkage thickness of the three-dimensional polymer matrix explains the formation of cavities where the ionic liquid prone to association can be concentrated. An excess of IL (6 mol) forms an individual phase, with the appearance of the second T_g_ corresponding to the glassy transition temperature of the IL itself.

### 3.3. TGA of NPEs

**Figure 3:**
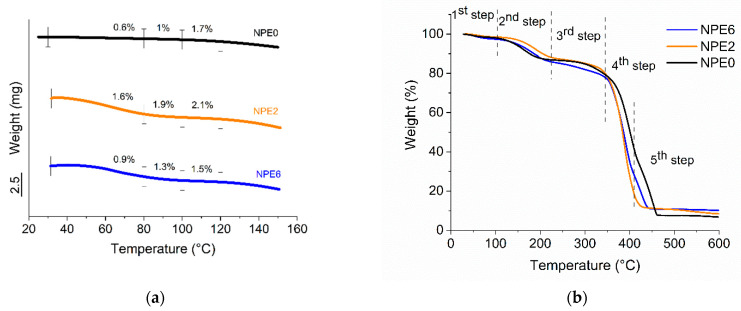
TGA—diagrams of all NPE compositions ranging from room temperature to 150 °C (**a**) up to 600 °C, where the 1st step is moisture loss, the 2nd step is EC loss, the 3rd step is the decay of salt LiBF_4_, the 4th step is ionic liquid loss, and the 5th step is the decay of polymer (**b**).

The TGA dependences of three NPE compositions are shown in Figure 3. Figure 3a shows that the polymer electrolytes are stable to 100 °C. The instrumental inaccuracy is the mass loss of 1%.

A slight weight loss may indicate a loss of moisture. Moisture accumulated during preparation for the study could get into the sample. The end of the first stage of the TGA diagram at 100 °C confirmed this.

Figure 3b shows the multistage character of sample weight loss. This explains the gradual loss of each component. The loss of ethylene carbonate (bp = 248 °C) occurs first. The ionic liquid, apparently, decomposes together with the polymer matrix at 390 °C. Titanium dioxide remains in the residue. Under extreme conditions, it is possible that TiO_2_ will insulate between the electrodes (if they are stable up to these temperatures).

### 3.4. Electrochemical Study of NPEs

The conductivity of the obtained NPE samples was measured at the first stage by the electrochemical impedance method in symmetrical SS//SS cells. Figure S3, ESI shows the Nyquist plots of the SS/NPE/SS cells at room temperature. The calculations of elements of equivalent circuit model are given in Table S3, ESI. The electrolyte resistance R decreases in the order NPE6 ˂ NPE2 ˂ NPE0. The value of CPE-P lies within 0.87÷0.77. Thus, it is possible to explain CPE by the capacitance of the electric double layer.

**Figure 4:**
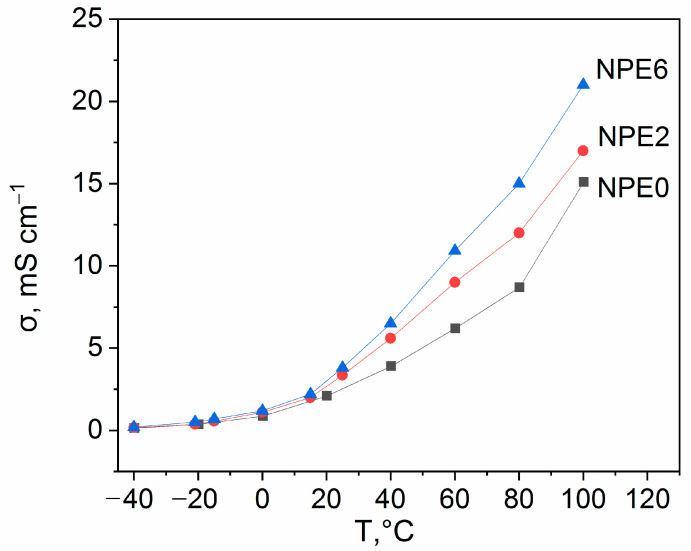
Temperatures dependences of the NPE conductivity.

The conductivity of all NPE samples was measured in the temperature range from −40 °C to 100 °C. The measurement results are given in Table S4, ESI and Figure 4.

The Arrhenius temperature dependence of the conductivity for all compositions (Figure S4, ESI) had a break in the temperature range from 15 to 25 °C, and, hence, the effective activation energy of conductivity was calculated in two ranges (Table 2). The calculations are shown in Figure S5, ESI for all three compositions of NPE.

Figure 4 and Table 2 show that the composition with 6 wt.% TiO_2_ has the highest conductivity among all the three studied thin film electrolytes.

The conductivity of NPE increases in the presence of TiO_2_, and the increase in conductivity is proportional to the content of TiO_2_. This effect can be explained by an increase in the degree of dissociation of the ion pairs EMIBF_4_ and LiBF_4_ in the near-surface layer of nanoparticles. With an increase in temperature, the ratio of the conductivity of NPE6 and NPE2 compositions increases; this indicates the difference in the near-surface layer at a high content of TiO_2_. Obviously, at a low content of TiO_2_, these layers around the nanoparticles are isolated to the greatest extent. An increase in conductivity with increasing temperature is associated with both an increase in the mobility of ions and an increase in the degree of dissociation. For the NPE2 composition, the activation energy of conduction in the high-temperature region is noticeably lower than the activation energy for the self-diffusion coefficients of all components of the composition (see below). Therefore, it can be concluded that an increase in the degree of dissociation is decisive. For NPE6 composition, the difference in these activation energies is leveled out. It can be assumed that the increase in conductivity with respect to NPE0 occurs due to the mobility of ions in adjacent near-surface layers.

### 3.5. High-Resolution NMR

**Figure 5:**
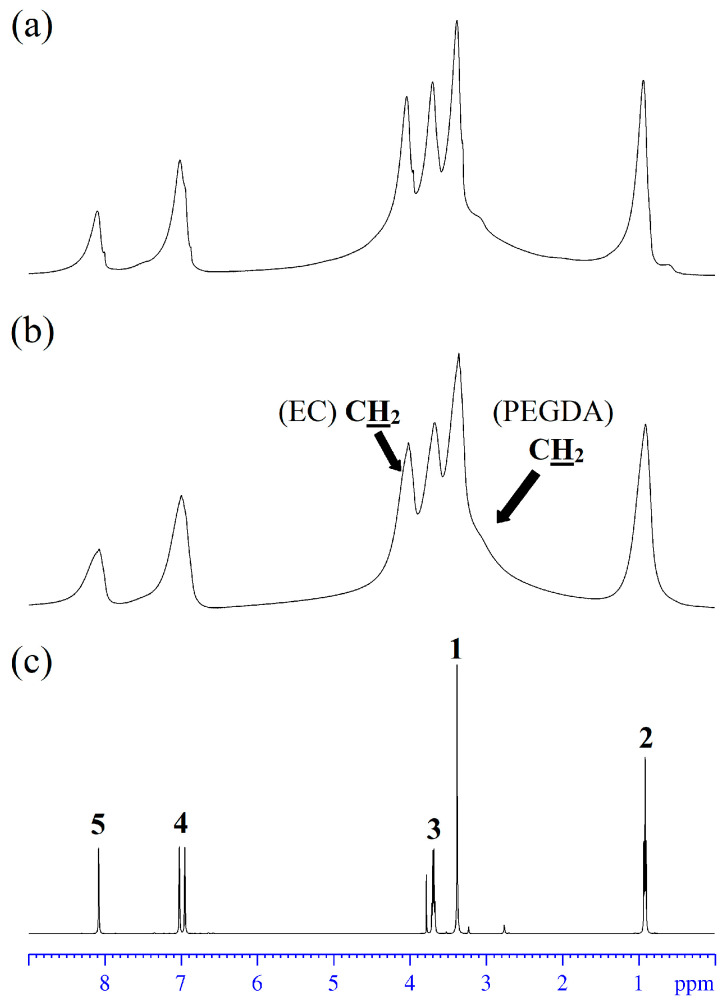
^1^H NMR spectra of the electrolytes (**a**) NPE2, (**b**) NPE6, and (**c**) ionic liquid EMIBF_4_ (atomic numbers at NMR peaks in (**c**) correspond the structure of EMI^+^ in Figure 1b).

**Figure 6:**
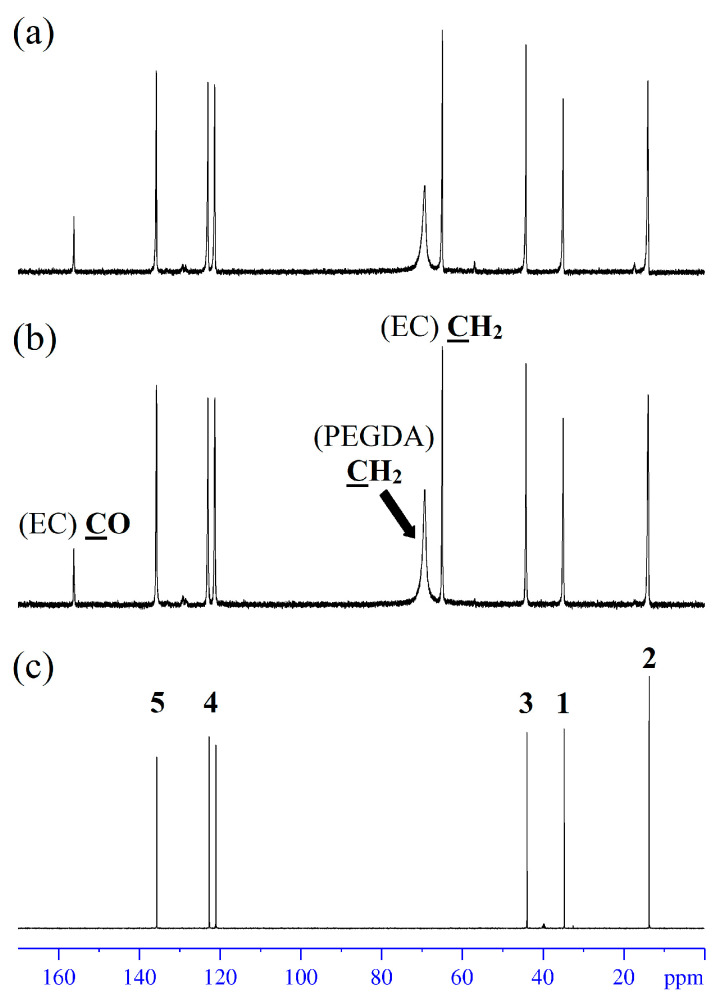
^13^C NMR spectra of the electrolytes (**a**) NPE2, (**b**) NPE6, and (**c**) ionic liquid EMIBF_4_ (atomic numbers at NMR peaks in (**c**) correspond the structure of EMI^+^ in Figure 1b).

The ^1^H and ^13^C NMR spectra of the NPE2 and NPE6 compositions compared with the initial ionic liquid EMIBF_4_ were recorded. The ^1^H and ^13^C NMR spectra are shown in Figure 5 and Figure 6, respectively. The ^1^H and ^13^C NMR spectra are identical for both NPE compositions. As can be seen, the signals in the ^1^H NMR spectra of the polymer electrolytes are significantly broader than those in pure EMIBF_4_. The signal of ethylene carbonate is also broadened (~4 ppm). The signal broadening is caused by the formation of a branched network polymer structure [28] formed by PEGDA, which considerably impedes the chaotic motion of EMIBF_4_ and EC. The ^1^H NMR spectrum of the electrolyte (Figure 5) exhibits a very broad signal from –O–CH_2_–CH_2_–O– of the polymer matrix with a maximum at ~3.5 ppm. This signal in the HSQC spectrum correlates with the ^13^C signal at 69.2 ppm in the ^13^C NMR spectrum (Figure 6).

The high–resolution ^7^Li, ^11^B, and ^19^F NMR spectra were also recorded (Figures S6–S8, ESI). The spectra show two phases, as in the case of experiments with self-diffusion.

### 3.6. Self-Diffusion Coefficients According to the PFG NMR Data

**Figure 7:**
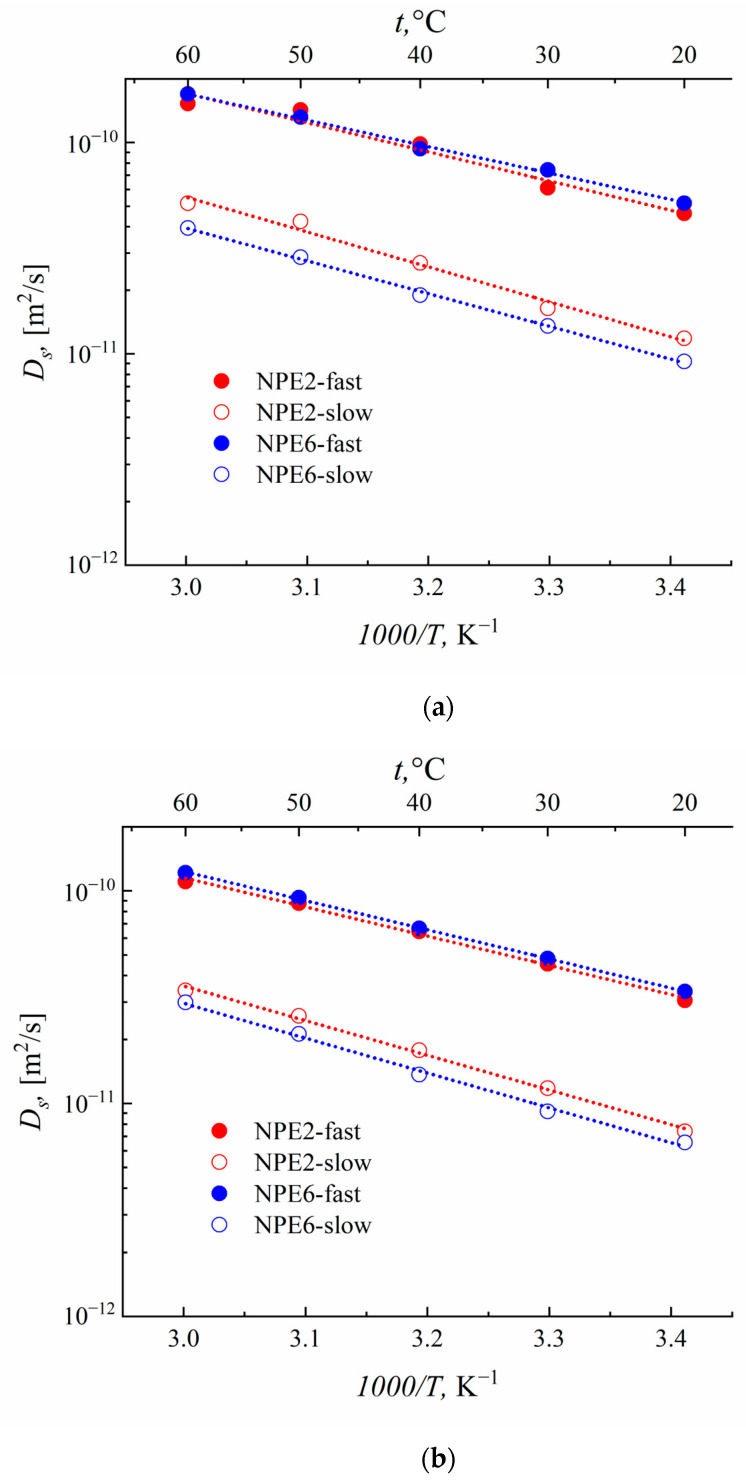
Temperature dependences of the diffusion coefficients of ^1^H for (**a**) EC and (**b**) IL with high- and low-phase populations.

**Figure 8:**
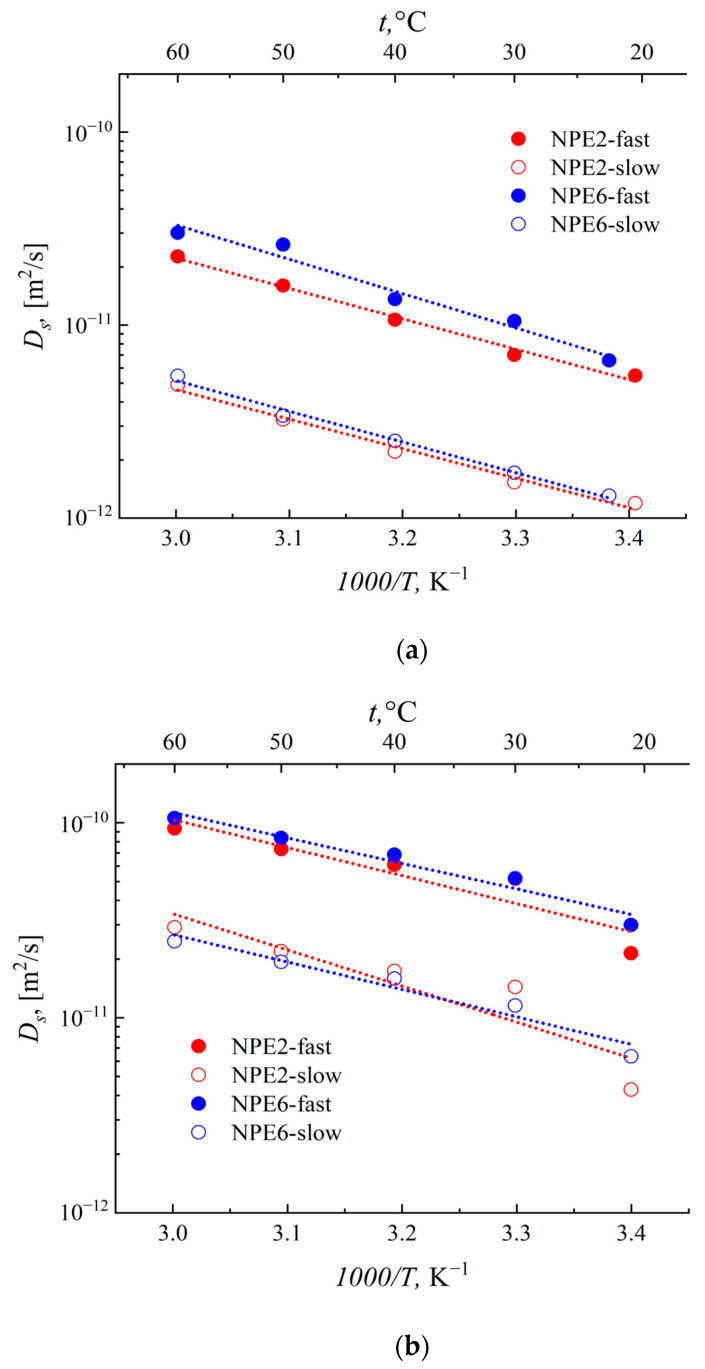
Temperature dependences of the diffusion coefficients of (**a**) ^7^Li and (**b**) ^19^F with fast- and slow-phase populations.

The SDC on ^1^H, ^7^Li, and ^19^F for the NPE2 and NPE6 compositions were measured by NMR with PGF. The diffusion decays on all nuclei of both compositions were not exponential (Figures S9 and S10). The measurements of the self-diffusion coefficients *D_s_* on ^1^H make it possible to determine the mobility of EMIBF_4_ and EC (analysis of the diffusion decay of the signals from the ionic liquid or ethylene carbonate solvent allows one to estimate their mobilities separately (Figure 7)). The *Ds* of ^7^Li corresponds to the mobility of lithium cations, and that of 19F corresponds to the mobility of the BF_4_^−^ anion (Figure 8).

The results of measuring *D_s_* for the NPE2 and NPE6 compositions are given in Table 3 and Table 4. The *D_s_* values for pure ionic liquid EMIBF_4_ are presented for comparison.

A different nuclei distribution over mobilities appears upon the addition of TiO_2_ nanoparticles. About 70% of all ^1^H, ^7^Li, and ^19^F nuclei have high SDC, which increases when going from 2 to 6 wt.% nanoparticles.

For the pure ionic liquid, the self-diffusion coefficient *D_s_* of ^19^F (mobility of BF_4_^−^) is lower than that of ^1^H (mobility of EMI^+^). The self-diffusion coefficients become close, (2.5–3.0) × 10^−11^ m^2^ s^−1^, when the anion and cation of the EMIBF_4_ ionic liquid are localized in the network of the polymer matrix. Upon the addition of TiO_2_ to 6 wt.%, the mobilities of the anion and cation of the ionic liquid are nearly equal. This also results in a slight increase in the *D_s_* of lithium cations.

The temperature dependences of the self-diffusion coefficients *D_s_* on ^1^H, ^7^Li, and ^19^F were measured in the range from 20 to 60 °C. Figure 7 and Figure 8 show the temperature dependences of the diffusion coefficients for ^7^Li, ^19^F, and ^1^H with high and low phase populations. These dependences are Arrhenius. The activation energies of diffusion were calculated (Table 5).

Table 5 shows that the activation energy of diffusion of the pure ionic liquid is ~20 kJmol^−1^ and that, in the polymer, the electrolyte composition is appreciably higher: 26–31 kJmol^−1^. As the TiO_2_ content increases, the activation energy of the diffusion of different NPE components differs in different ways. The E_a_ values of the EC solvent molecules and BF_4_^−^ anions decrease. The activation energy of the diffusion of lithium increases, while that of the ionic liquid remains unchanged.

From the effect of an increase in the degree of dissociation of ion pairs EMIBF_4_ and LiBF_4_ in the near-surface layer of nanoparticles, one can interpret the data on the influence of the TiO_2_ content on the activation energies of the self-diffusion coefficients of various components (Table 5). An increase in the contribution of the ionic components, which depend less on temperature than the contribution of the neutral components, should effectively lead to a decrease in the activation energy. Correspondingly, this occurs for nuclei of all types, with the exception of ^7^Li. Bulky imidazolium ions seem to make the smallest contribution to conductivity, so the effect of^1^H EMI^+^ is the smallest. The only exception is the ^7^Li nucleus, for which an increase in activation energy is observed. However, in this case, there is a noticeable decrease in the transfer number (by one and a half times). Therefore, on the contrary, the contribution from the mobility of the Li^+^ ion only decreases and the effect is reversed.

### 3.7. Theoretical Analysis

Earlier in Reference [36], for a similar electrolyte system but with the introduction of SiO_2_ nanoparticles, only one mobility coefficient was observed for all of the components of the system. In addition, it was shown that the electrolyte based on SiO_2_, with the same composition of all other components (ionic liquid, salt, polymer, and EC), had conductivity with respect to lithium ions sufficient for its use in lithium–organic batteries. However, this conductivity was not achieved for systems with TiO_2_. In order to further modify the electrolyte system, it is necessary to understand the differences in the conductivity mechanisms involving different types of nanoparticles. To reveal the reasons for the presence of two types of molecular mobility in systems with TiO_2_ nanoparticles, quantum chemical calculations were carried out using the density functional theory method.

In a previous work [36], a SiO_2_ nanoparticle was modeled by a Si_17_O_28_(OH)_12_ cluster. When studying its interaction with electrolyte components, it was found that the formation of the adsorption complex {Si_17_O_28_(OH)_12_(Li BF_4_)_2_ (EMI BF_4_)_2_} reduces the energy of the system by 1.47 eV. This complex contains five hydrogen bonds of the surface –OH groups with the F atoms of the BF_4_^−^ anion in the range 1.98–2.17 Å.

**Figure 9:**
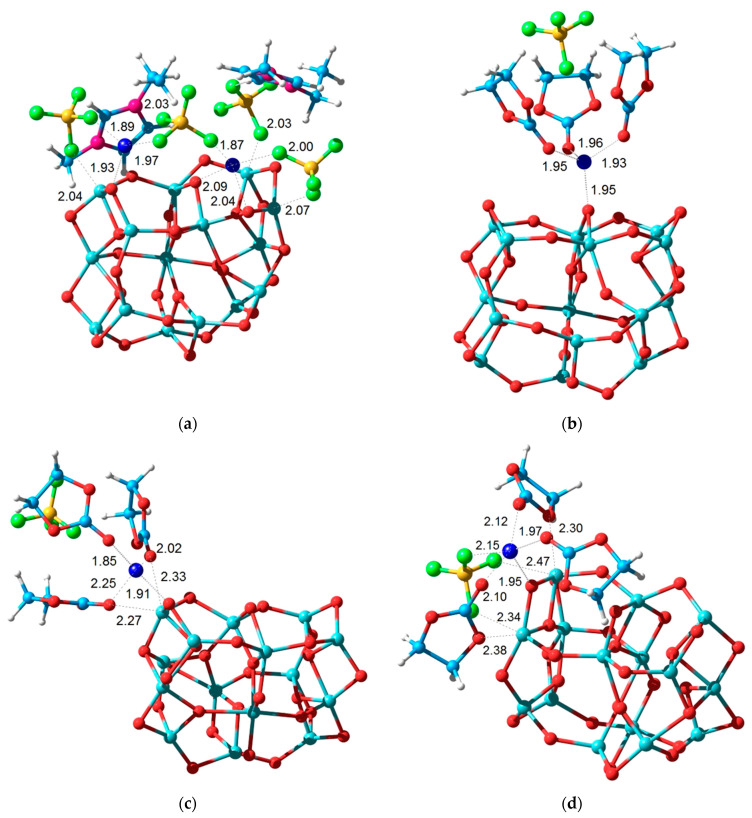
Calculated structure of the surface complexes {Ti_15_O_30_ (Li BF_4_)_2_ (EMI BF_4_)_2_} (**a**) and {Ti_15_O_30_ (Li BF_4_)(EC)_3_} isomers (**b**–**d**).

In this work, a TiO_2_ nanoparticle was simulated by a Ti_15_O_30_ cluster (Figure 9a), which lacks surface –OH groups according to IR spectroscopy data (Figure S11, ESI). In the crystal structure of TiO_2_, Ti atoms have a coordination number of 6. There are few such atoms in the model cluster, and most Ti atoms have a coordination number of 5 or 4, since they are located on the surface layer with broken Ti–O bonds.

According to the calculation data, the addition of a water molecule to the Ti_15_O_30_ cluster supplies an energy gain of 1.34 eV, which is noticeably lower than the energy of H_2_O addition to the Si_17_O_34_ cluster: 2.69 eV. This difference is responsible for the observed hydration of the surface of SiO_2_ nanoparticles.

The presence of coordinatively unsaturated Ti atoms on the surface of a nanoparticle leads to several effects. They are able to form additional short contacts both with the O atoms of the solvent molecules (Figure 9c) and with the F atoms of the BF_4_^−^ anions included in the first coordination sphere of the Li^+^ ions (Figure 9d). As a result, there is a significant increase in the energy of interaction with the solvated ion pair {Li^+^BF_4_^−^(EC)_3_}, up to 1.50 eV and 1.77 eV, respectively. In the absence of such interactions, the energy of formation of a surface complex involving the bridging O atom is 0.63 eV (Figure 9b) and is practically the same as for the SiO_2_ nanoparticle.

A noticeable increase in the energy of formation of the {Ti_15_O_30_(Li BF_4_)_2_ (EMI BF_4_)_2_} surface complex up to 3.24 eV compared to the SiO_2_ analog occurs due to the formation of three short Ti–F coordination bonds with BF_4_^−^ anions in the range 2.03–2.07 Å. In fact, this indicates the presence of the effect of strong chemisorption of anions from the solution, which, in turn, will retain counterions around them due to Coulomb and coordination interactions. Thus, an intermediate stage appears in the ion transport through the electrolyte when they are retained for some time in the near-surface layer of nanoparticles. This delay is directly manifested in the study of samples with TiO_2_ nanoparticles by pulsed field gradient NMR, which showed the presence of two types of molecular mobility with very different self-diffusion coefficients.

These molecular models correspond to a stoichiometric molar ratio of LiBF_4_:EC = 1:3. Obviously, when the relative EC content is increased to 1:4, the additional EC molecule will not enter the chemisorbed complex. Thus, the EC content in the electrolyte will increase, which will favour better mobility of Li^+^ ions.

New samples of thin film NPEs* with a molar content of PEGDA:LiBF_4_:EMIBF_4_:EC = 1:1:6:4 components with TiO_2_ content of 0, 2 and 6 wt.% were obtained. The ionic conductivity of the obtained films without TiO_2_ was 4.8 mS cm^−1^, while for the samples with 2 and 6 wt.% TiO_2_ it was the same and equaled 4.4 mS cm^−1^.

**Figure 10:**
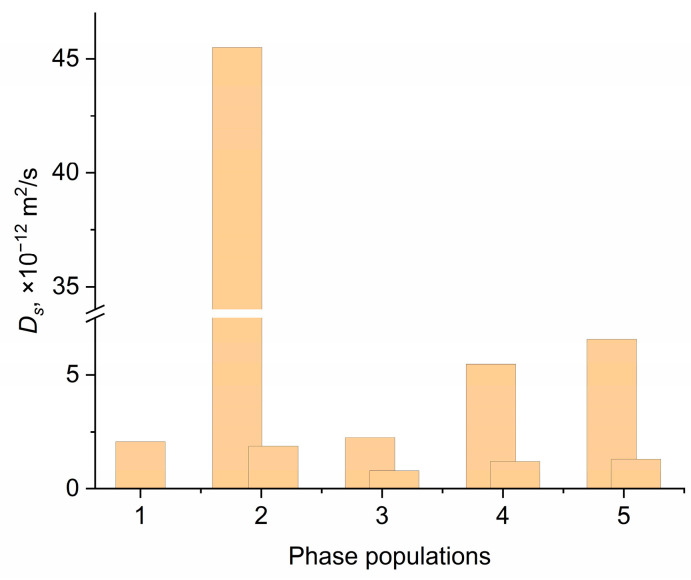
Dependence of self-diffusion coefficients on ^7^Li nuclei for samples (1) NPE0* (*p* = 1), (2) NPE2* (*p*_1_ = 0.1, *p*_2_ = 0.9), (3) NPE6* (*p*_1_ = 0.7, *p*_2_ = 0.3), (4) NPE2 (*p*_1_ = 0.7, *p*_2_ = 0.3), (5) NPE6 (*p*_1_ = 0.7, *p*_2_ = 0.3).

From the PFG NMR data it can be seen (Figure 10) that, in the case of NPE2*, the mobility of lithium increases. This indicates the formation of a highly mobile lithium ion.

### 3.8. Electrochemical Study of NPEs in Li//LiFePO_4_ Cells

In this work, battery prototypes with a cathode based on LiFePO_4_ (LPF) were assembled. Films composed of NPE* with a molar content of PEGDA:LiBF_4_:EMIBF_4_:EC = 1:1:6:4 components with TiO_2_ contents of 0, 2 and 6 wt.% were used for the assembly. The concept of “liquid therapy” for cell assembly has been used similarly [37]. A 1 M solution of LiTFSI in DOL/DME was used to wet the electrode surface [37]. This approach can significantly reduce the resistance at the NPE/electrode interface, as shown in our previous work [37].

**Figure 11:**
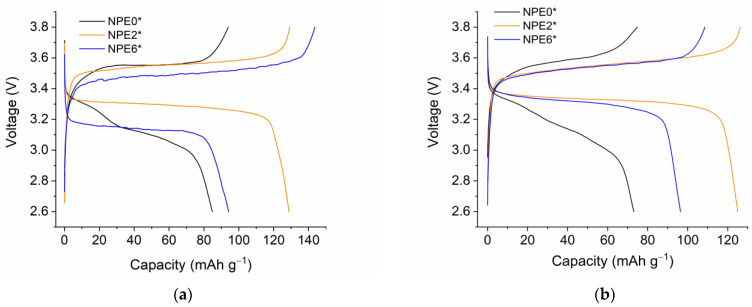
Charge-discharge profiles of Li/NPE/LiFePO_4_ cells for the second cycle (**a**) and the 10th cycle (**b**).

The charge-discharge profiles of Li/NPE/LFP are shown in Figure 11 for the second and tenth cycles.

During the discharge process, lithium is intercalated into the olivine structure, and during the charging process, lithium is deintercalated from the inorganic cathode. The lithium cation then passes through the membrane NPE volume and is deposited on the lithium metal anode.

Figure 11 shows that the discharge plateau is more pronounced in the presence of TiO_2_ nanoparticles compared to the original NPE0* composition. Thus, TiO_2_ nanoparticles improve the process of lithium intercalation into the cathode material. Apparently, this is due to a decrease in the resistance of the electrode reaction, which follows from the difference between the charge plateau and discharge plateau potentials. Figure 11 shows that this value is smaller for NPE2* (0.24 V, 0.21 V for the second and tenth cycle, respectively) than for NPE6* (0.35 V, 0.22 V for the second and tenth cycle, respectively). For NPE0*, the discharge curve does not have a pronounced horizontal plateau. If we use the estimated potential value for the middle of the descending plateau, it is found that, in the absence of nanoparticles, the potential difference between the discharge plateaus for the second and tenth cycles for the cell with NPE0* increases significantly to 0.45V and 0.53V, respectively.

**Figure 12:**
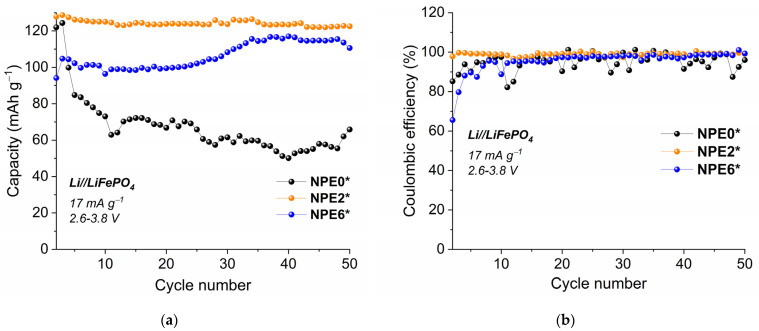
Cycling performance of the Li/NPE/LiFePO_4_ cells (**a**) and dependence of the Coulomb efficiency on the cycle number (**b**) at current density 17 mAh g^−1^ (C/10) in a range of 2.6–3.8 V.

Since the nanocomposite electrolyte is a “rigid system” in which the nanoparticles are fixed by a three-dimensional polymer matrix, they are also firmly anchored in the near-surface layer at the electrode. Then, a new lithium cation transport channel appears for the nanocomposite through the surface layer of nanoparticles, which is quasi-reversible. Therefore, for the cell with NPE0*, there is no horizontal plateau (Figure 11), and an unsatisfactory cyclicity of the cell is observed (Figure 12a) due to the accumulation of irreversible effects in the absence of nanoparticles. The contribution of lithium-ion transport through the near-surface layers of nanoparticles can be expected to increase with increasing nanoparticle content. According to the results of measuring the self-diffusion coefficients (Figure 8a), this is true for NPE2 and NPE6 compositions. However, as the EC content increases for NPE2* and NPE6* compositions, the mobility ratio becomes reversed (Figure 10), with a giant increase in the fast self-diffusion coefficient of lithium ions. Understanding the causes of this phenomenon requires special research, which is beyond the scope of this article.

The cell cycling performance is shown in Figure 12a, and the Coulomb efficiency is shown in Figure 12b.

It can be seen from Figure 12 that the Li/NPE2*/LiFePO_4_ cell has the best cycling performance in terms and Coulomb efficiency.

Testing of Li/NPE*/LFP cells (Figure 12) show that the NPE2* composition increases the reversibility of the electrode reaction, in contrast to the electrolyte system without TiO_2_. NPE2* has the highest discharge capacity of 124 mAh g^−1^ with a Coulomb efficiency of 99%. For the NPE6*, the capacity is lower at 104 mAh g^−1^ and the efficiency is ~98%. Using the NPE0* electrolyte, the capacity ranges from 121 to 123 mAh g^−1^ for 1–2 cycles then gradually decreases to 65 mAh g^−1^ (Figure 12). For cells with nanocomposite electrolytes, capacitance and Coulomb efficiency correlate with lithium ion mobility.

Figure S12 (ESI) shows the dependence of the specific capacity on the rate of the charge-discharge current in the C/10 to C/2 range. The current rate capability of the cells has shown that, for different NPE, the capacity is very different when the speed changes from C/10 to C/2. For NPE0*, it drops by 4 times. Thus, the NPE2-6* structure is optimal for the transport of Li ions.

Table 6 summaries the selected data for solid-state batteries presented in the literature [38,39,40,41,42] which contain electrolytes of nanocomposites or similar materials and the results of this work for quick reference.

The closest in terms of the method of obtaining a polymer electrolyte are references [38,39]. Poly(ethylene glycol) methyl ether acrylate (PEGMEA) is used as the monomers with dibenzoyl peroxide initiator for in situ polymerization of solutions into polyethylene (PE) separator [38,39].

Review [40] describes the lithium phosphorus oxygen nitrogen (LiPON) as solid electrolyte with poor interface with the electrode. There are many preparation methods of the best LiPON.

In reference [41], iron–nickel–cobalt trimetal Prussian blue analogue (PBA) nanocubes are filled into electrospun polyacrylonitrile (PAN)-based membranes which had good thermal stability, high porosity and liquid electrolyte uptake.

Work [42] describes an approach similar to ours, creating a transitional protective layer on a lithium electrode. A quasi-ionic liquid-based polymer electrolyte layer was attached to the interface of 3D lithium–boron (LiB) anode by a casting method. The polymer electrolyte consisted of LiTFSI/PVDF-HFP.

Table 6 shows that the nanocomposite electrolytes obtained in this work have a wider range of operating temperatures from −40 to 100 °C, in which the ionic conductivity has high values.

## 4. Conclusions

The new nanocomposite polymer gel electrolytes based on the network matrix of polyethylene glycol diacrylate were prepared. This matrix was immobilized by the ionic liquid 1–ethyl–3–methylimidazolium tetrafluoroborate, the salt LiBF_4_, a minor amount of ethylene carbonate, and TiO_2_ nanopowder (d~21 nm). Thin-film polymer electrolytes with a high conductivity of 4.8 mS cm^−1^ at room temperature were prepared. The operating temperature ranged from −40 to 100 °C. The synthesized films had good thermal stability and high conductivity in the given temperature range. The obtained systems have advantages over electrolytes for solid-state batteries known from the literature [38,39,40,41,42].

The ion and molecular transport in these systems in the presence of TiO_2_ nanoparticles was studied by the NMR method. All particles were found to possess two diffusion coefficients for fast and slow mobility.

This effect, according to quantum chemical modeling, is the result of the strong chemisorption of anions BF_4_^−^ on the TiO_2_ surface, which, in turn, will retain counterions around them due to Coulomb and coordination interactions. Thus, an intermediate stage appears in the transport of some ions through the electrolyte when they are retained for some time in the near-surface layer of nanoparticles.

The fundamental knowledge obtained about the mechanism of interaction between TiO_2_ nanoparticles and ions made it possible to modify the composition by adding an extra mole of ethylene carbonate. It leads to an increase of an order of magnitude of the self-diffusion coefficient of lithium for the sample with 2 wt.% TiO_2_.

This design of the NPE composition made it possible to obtain promising thermostable polymer electrolytes for safe lithium batteries with LiFePO_4_ cathode.

## Figures and Tables

**Table 1:** Compositions of the nanocomposite polymer gel electrolytes based on the ionic liquid.

No.	Content	PEGDA	LiBF_4_	EMIBF_4_	EC	TiO_2_	PB
NPE0	mol	1	1	6	3		
	wt.%	27	4	55	12	0	1
NPE2	mol	1	1	6	3		
	wt.%	30	4	51	12	2	1
NPE6	mol	1	1	6	3		
	wt.%	29	4	49	11	6	1

**Table 2:** Conductivity of the NPE and the effective activation energy.

No.	σ, mS cm^−1^ (25 °C)	*Ea*, kJ mol^−1^	
		−40 to +15 °C	25 to 100 °C
NPE0	2.1	25.4 ± 0.8	21.2 ± 1.8
NPE2	3.4	25.5 ± 1.3	19.4 ± 1.2
NPE6	3.8	24.1 ± 0.5	20.7 ± 1.3

**Table 3:** Self-diffusion coefficients of ^7^Li and ^19^F, where *p* is the phase population at 24 °C.

Sample	*Ds*, m^2^s^−1^ (10^−12^)			
	^7^Li (*p*)		^19^F (*p*)	
NPE2	5.47 (0.7)	1.19 (0.3)	21.4 (0.7)	4.29 (0.3)
NPE6	6.56 (0.7)	1.31 (0.3)	29.9 (0.7)	6.35 (0.3)
EMIBF_4_	—		38.5 (1)	

**Table 4:** Self-diffusion coefficients of ^1^H nucleus, where *p* is the phase population, at 24 °C.

Sample	*Ds*, m^2^s^−1^ (10^−12^)			
	^1^H (*p*), EMI^+^		^1^H (*p*), EC	
NPE2	30.6 (0.7)	7.40 (0.3)	46.1 (0.7)	11.8 (0.3)
NPE6	33.6 (0.7)	6.56 (0.3)	51.7 (0.7)	9.22 (0.3)
EMIBF_4_	51.5 (1)		—	

**Table 5:** Activation energy of diffusion for ^1^H, ^7^Li, and ^19^F.

Sample	Ea, kJmol^−1^ (eV)							
	^1^H_EMI^+^		^1^H_EC		^7^Li		^19^F	
	fast	slow	fast	slow	fast	slow	fast	Slow
NPE2	26.3 (0.27)	31.2 (0.32)	26.4 (0.27)	31.6 (0.33)	30.1 (0.31)	29.2 (0.30)	27.5 (0.28)	35.4 (0.37)
NPE6	26.2 (0.27)	31.3 (0.32)	23.9 (0.25)	29.6 (0.31)	34.0 (0.35)	30.4 (0.32)	25.0 (0.26)	26.9 (0.28)
EMIBF_4_	20.9 (0.22)				—		27.0 (0.28)	

**Table 6:** The polymer and composite electrolytes for solid-state batteries.

Electrolyte	Conductivity, S cm^−1^	Cells Performance	Reference
PEGMEA/LiTFSI + 1 M LiPF_6_ in EC/DMC/EMC (1/1/1, *v*/*v*/*v*) (PE—separator)	10^−5^ (20 °C)	Li//LiFePO_4_ *	[38]
	2 × 10^−4^ (60 °C)	158 mAh g^−1^ (80 °C)	
PEGMEA/LiTFSI (PE—separator)	1.1 × 10^−4^ (20 °C)	Li//LiFePO_4_	[39]
		149 mAh g^−1^ (60 °C)	
PEGDA, LiBF_4_, EMIBF_4_, EC, and TiO_2_ nanopowder	2 × 10^−4^ (−40 °C)	Li//LiFePO_4_	This work
	1 × 10^−3^ (0 °C)	124 mAh g^−1^ (20 °C)	
	4.8 × 10^−3^ (20 °C)		
	2.1 × 10^−2^ (100 °C)		
LiPON solid electrolytes	2 × 10^−6^	—	[40]
PAN—PBA composite membrane with liquid electrolyte	2.7 × 10^−3^	Li//LiFePO_4_	[41]
		152 mAh g^−1^	
LiTFSI/PVDF-HFP on LiB anode	—	Li//NMC83 **	[42]
1 M LiPF_6_ in EMC/DMC/FEC (7:2:1 by volume)		203 mAh g^−1^	

* LiFePO_4_ conclude PVDF/LiClO_4_; ** NMC83 (LiNi_0.83_Mn_0.06_Co_0.11_O_2_).

## Data Availability

Not applicable.

## Supplementary Materials

The following supporting information can be downloaded at: https://www.mdpi.com/article/10.3390/membranes13090776/s1, Figure S1. (a) SEM image of the TiO_2_ nanopowder; (b) photographs of the NPE6* film; (c) optical images of the NPE0 film surface and (d) NPE6 film surface; Table S1. Compositions of the nanocomposite polymer gel electrolytes; Figure S2. Dependence of the NPE conductivity on the content of TiO_2_ nanoparticles; Table S2. Glass transition temperature of NPE (according to the DSC data); Figure S3. Nyquist plots of the SS/NPE/SS cell at room temperature for (a) NPE0, (b) NPE2, (c) NPE6 and the equivalent circuit models, where R is the electrolyte resistance, CPE is the constant phase element, (d) the high frequency region of the Nyquist plots for all NPEs; Table S3. Calculation of elements of equivalent circuit model; Table S4. Conductivity of the nanocomposite polymer gel electrolytes based on ionic liquid EMIBF_4_; Figure S4. Arrhenius temperature dependences of the NPE conductivities; Figure S5. Calculation of the effective conduction activation energy for NPE0 (a,b), NPE2 (c,d) and NPE6 (e,f) in two temperature ranges: from −40 to 25 °C (a,c,e) and from 40 to 100 °C (b,d,f); Figure S6. 7Li NMR spectra of the electrolytes (a) NPE2 and (b) NPE6; Figure S7. ^11^B NMR spectra of electrolytes (a) NPE2, (b) NPE6, and (c) ionic liquid EMIBF_4_; Figure S8. ^19^F NMR spectra of the electrolytes (a) NPE2, (b) NPE6, and (c) ionic liquid EMIBF_4_; Figure S9. Diffusion decays of NPE2 and NPE6 on ^1^H nuclei (a) EC and (b) EMIBF_4_; Figure S10. Diffusion decays of NPE2 and NPE6 on (a) ^7^Li and (b) ^19^F nuclei; Figure S11. FTIR spectra of the TiO_2_ and SiO_2_ nanoparticles; Figure S12. Current rate capability of the Li/NPE/LiFePO_4_ cells in the C/10 to C/2 range.
