# Supplementary material for: Nanocomposite Polymer Gel Electrolyte Based on TiO2 Nanoparticles for Lithium Batteries
Source: Membranes (Basel). 2023 Sep 1;13(9):776. doi: 10.3390/membranes13090776 (PMC10536963; doi:10.3390/membranes13090776)
Supplement: Supplementary file 1 [file membranes-13-00776-s001.zip › membranes-2560692-supplementary.pdf]

Electronic supplementary information

Nanocomposite polymer gel electrolyte based on TiO_2 nanoparticles for lithium batteries

Nikita A. Slesarenko, Alexander V. Chernyak, Kyunsylu G. Khatmullina, Guzaliya R. Baymuratova, Alena V. Yudina, Galiya Z. Tulibaeva, Alexander F. Shestakov, Vitaly I. Volkov, and Olga V. Yarmolenko

Table of Contents

Figure S1. **(a)** SEM image of the TiO_2 nanopowder; **(b)** photographs of the NPE6* film; **(c)** optical images of the NPE0 film surface and **(d)** NPE6 film surface 2

Table S1. Compositions of the nanocomposite polymer gel electrolytes 3

Figure S2. Dependence of the NPE conductivity on the content of TiO_2 nanoparticles.........3

Table S2. Glass transition temperature of NPE (according to the DSC data) 4

Figure S3. Nyquist plots of the SS/NPE/SS cell at room temperature for (a) NPE0, (b) NPE2, (c) NPE6 and the equivalent circuit models, where R is the electrolyte resistance, CPE is the constant phase element, (d) the high frequency region of the Nyquist plots for all NPEs. 5

Table S3. Calculation of elements of equivalent circuit model...6

Table S4. Conductivity of the nanocomposite polymer gel electrolytes based on the ionic liquid $EMIBF_4$ 6

Figure S4. Arrhenius temperature dependences of the NPE conductivities 6

Figure S5. Calculation of the effective conduction activation energy for NPE0 **(a, b)**, NPE2 **(c, d)** and NPE6 **(e, f)** in two temperature ranges: from −40 to 25 °C **(a, c, e)** and from 40 to 100 °C **(b, d, f)** 7

Figure S6. ^{7}Li NMR spectra of the electrolytes **(a)** NPE2 and **(b)** NPE6 8

Figure S7. ^{11}B NMR spectra of the electrolytes **(a)** NPE2, **(b)** NPE6 and **(c)** ionic liquid $EMIBF_4$ 8

Figure S8. ^{19}F NMR spectra of the electrolytes **(a)** NPE2, **(b)** NPE6 and **(c)** ionic liquid $EMIBF_4$ 9

Figure S9. Diffusion decays of NPE2 and NPE6 on ^{1}H nuclei for **(a)** EC and **(b)** $EMIBF_4$ ...10

Figure S10. Diffusion decays of NPE2 and NPE6 on **(a)** ^{7}Li and **(b)** ^{19}F 11

Figure S11. FTIR spectra of the TiO_2 and SiO_2 nanoparticles 12

Figure S12. Current rate capability of the Li // $LiFePO_4$ cells in the C/10 to C/2 range: **(a)** NPE0*; **(b)** NPE2*; **(c)** NPE6* 13

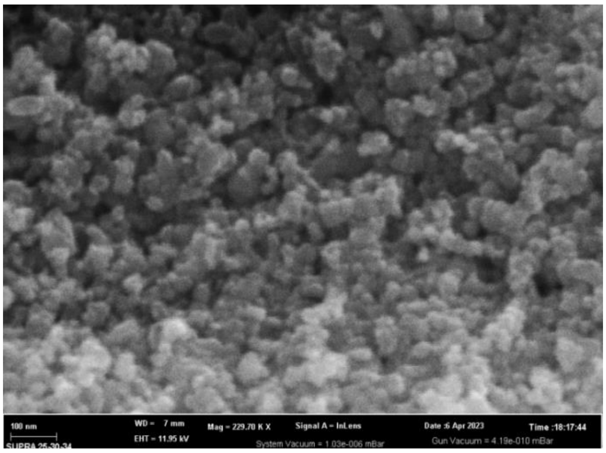

(a)

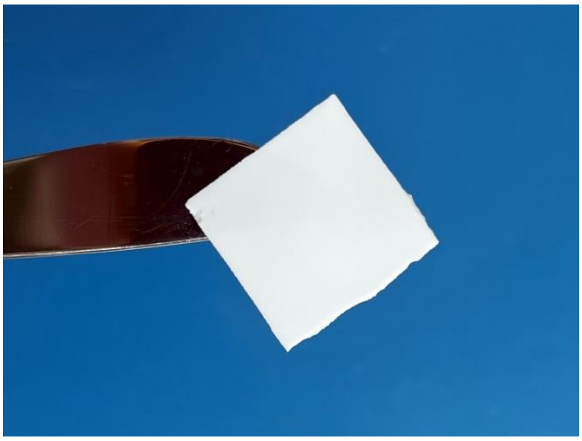

(b)

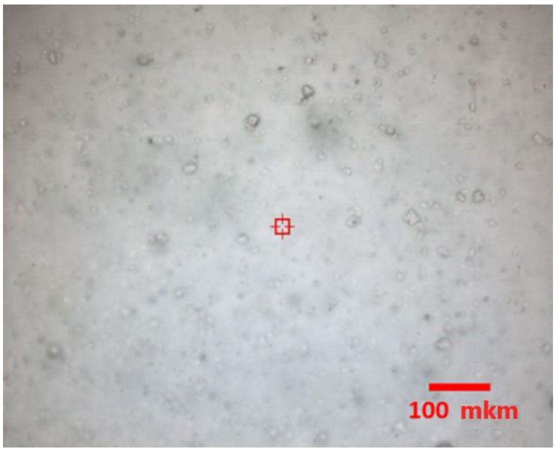

(c)

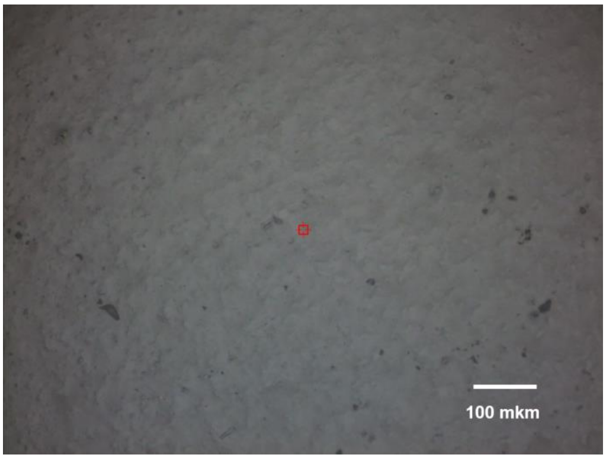

(d)

Figure S1. (a) SEM image of the TiO_2 nanopowder; **(b)** photographs of the NPE6* film; **(c)** optical images of the NPE0 film surface and **(d)** NPE6 film surface

SEM image of the TiO_2 nanopowder wase obtained using a ZEISS LEO SUPRA 25 scanning autoemission electron microscope.

The optical images of the NPE film surfaces were obtained using a DRX—2 microscope (Thermo Fisher, USA) with 10× objective with an NA of 0.50.

Table S1. Compositions of the nanocomposite polymer gel electrolytes

No.	PEGDA, wt.%	$LiBF_4$:EC (1:3 mol), wt.%	TiO_2, wt.%	PB, wt.%
1	30	69	0	1
2	30	67	2	1
3	30	65	4	1
4	30	63	6	1
5	30	61	8	1

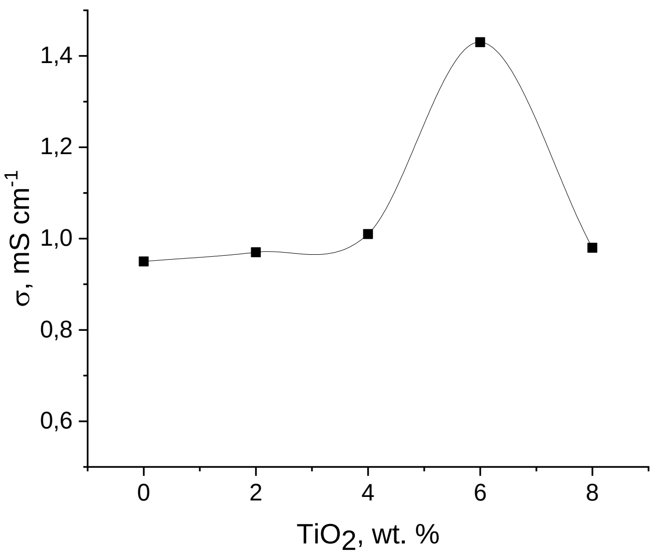

Figure S2. Dependence of the NPE conductivity on the content of TiO_2 nanoparticles

Table S2. Glass transition temperature of NPE (according to the DSC data)

№	T_{01}, °C	T_{g1}, °C	ΔT_1, °C	T_{g2}, °C	ΔT_2, °C
NPE0	−78.0	−61.5	32.9	−102.3	4.3
NPE2	−78.9	−66.0	22.0	−98.6	3.9
NPE6	−83.0	−66.8	28.4	−98.4	3.5
$EMIBF_4$	—	—	—	−103.0	4.0

Note. T_{01} is the onset of the relaxation transition; T_{g1} is the temperature of the first relaxation transition (relaxation transition of the cross—linked polymer matrix); ΔT_1 is the range of the first relaxation transition; T_{g2} is the glass transition temperature of the second relaxation transition); ΔT_2 is the range of the second relaxation transition.

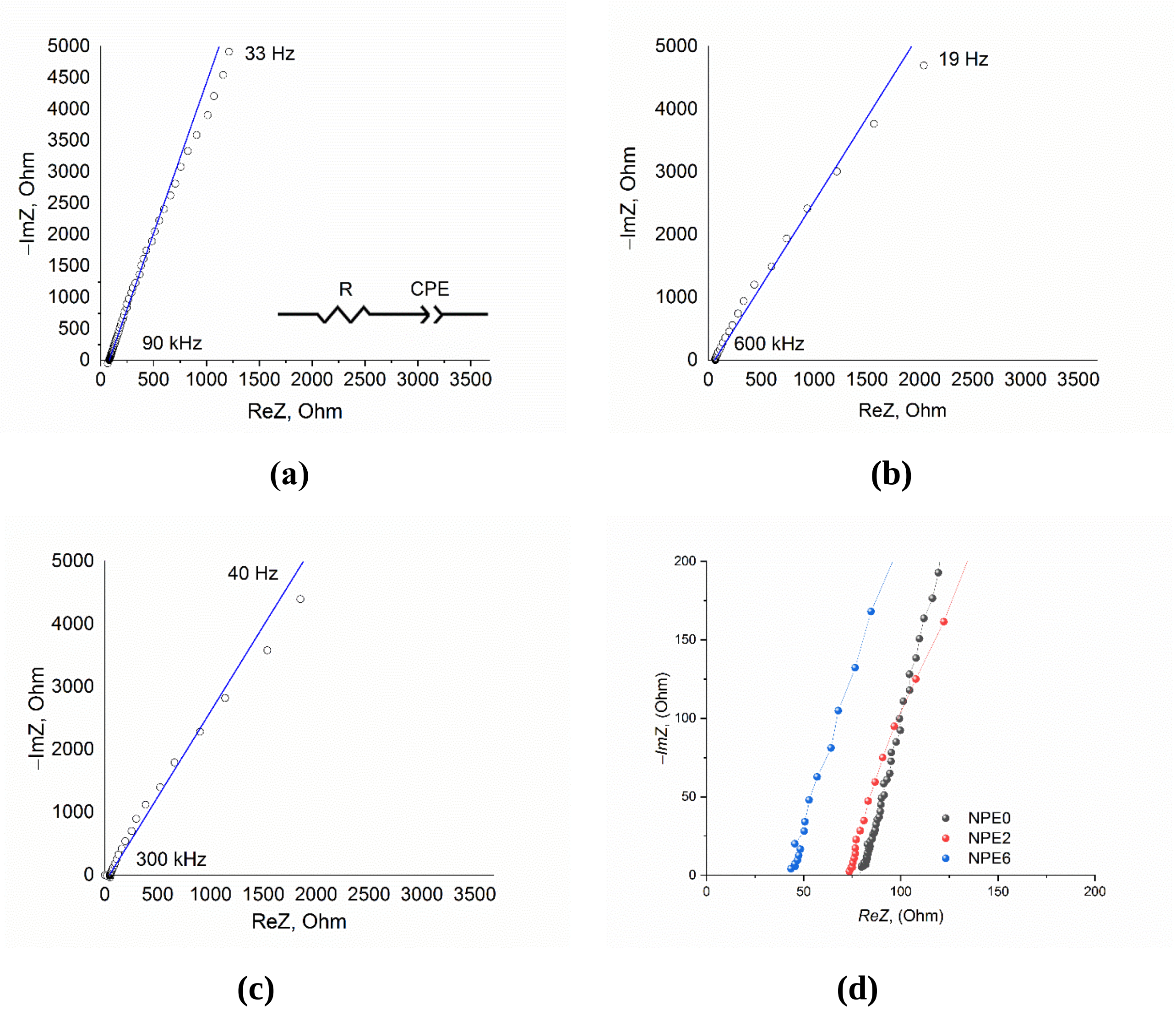

Figure S3. Nyquist plots of the SS/NPE/SS cell at room temperature for (a) NPE0, (b) NPE2, (c) NPE6 and the equivalent circuit models, where R is the electrolyte resistance, CPE is the constant phase element, (d) the high frequency region of the Nyquist plots for all NPEs.

Table S3. Calculation of elements of equivalent circuit model

Equivalent circuit element	SS/NPE/SS cells		
	NPE0	NPE2	NPE6
R, Ohm	79.6	57.4	42.8
CPE—T	1.9×10^{-6}	2.1×10^{-6}	2.3×10^{-6}
CPE—P	0.87	0.77	0.78

Table S4. Conductivity of the nanocomposite polymer gel electrolytes based on the ionic liquid $EMIBF_4$

T, °C	Conductivity, mS cm^{-1}		
	NPE0	NPE2	NPE6
—40	0.1	0.2	0.2
—21	0.4	0.4	0.5
—15	—	0.6	0.7
0	0.9	1.1	1.2
15	—	2.0	2.2
25	2,1	3.4	3.8
40	3.9	5.6	6.5
60	6.2	9.0	10.9
80	8.7	12.0	15.0
100	15.1	17.0	21.0

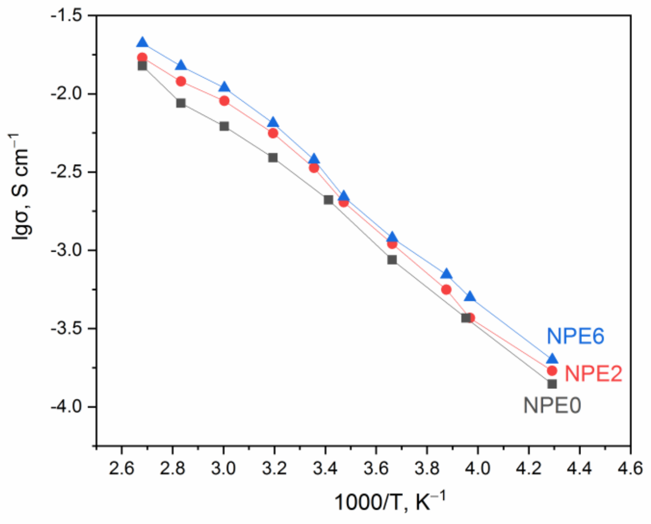

Figure S4. Arrhenius temperature dependences of the NPE conductivities

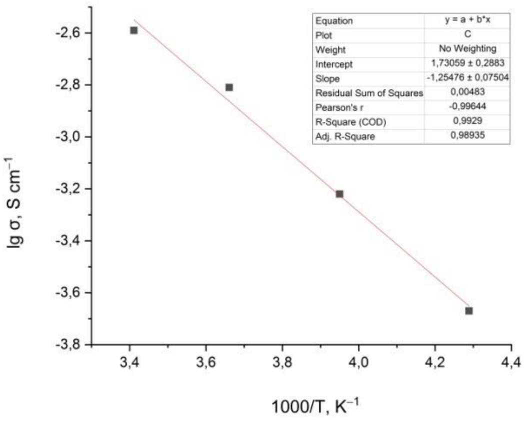

(a)

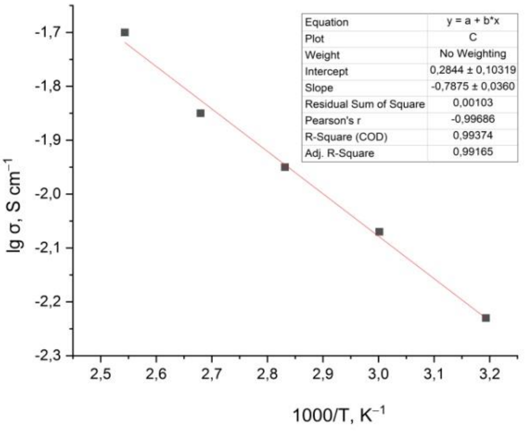

(b)

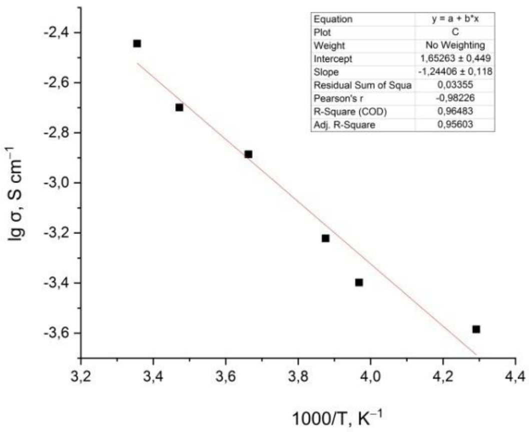

(c)

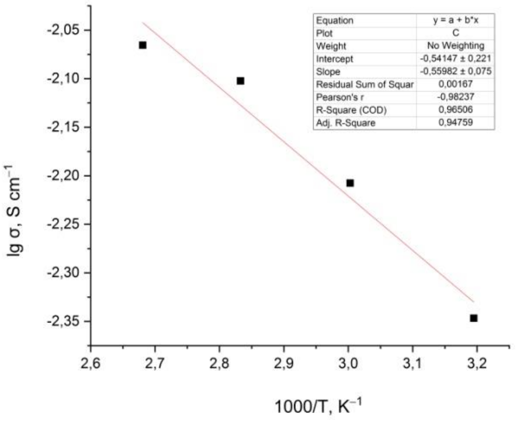

(d)

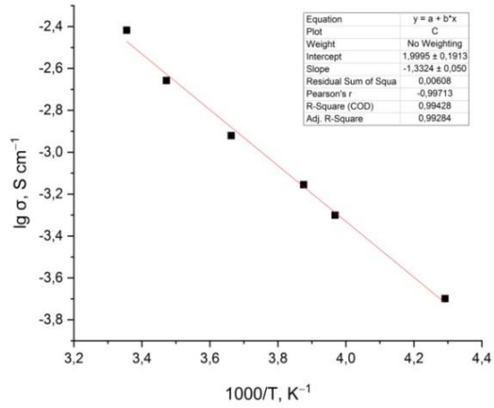

(e)

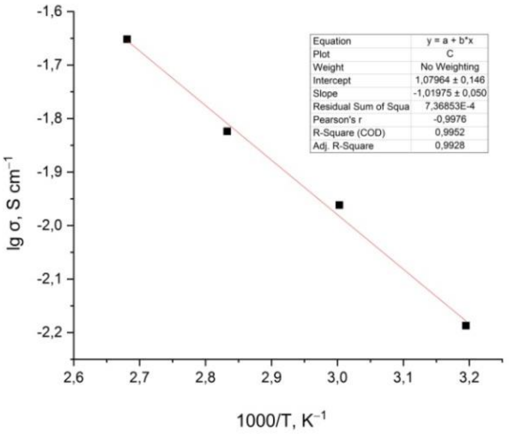

(f)

Figure S5. Calculation of the effective conduction activation energy for NPE0 **(a, b)**, NPE2 **(c, d)** and NPE6 **(e, f)** in two temperature ranges: from —40 to 25 °C **(a, c, e)** and from 40 to 100 °C **(b, d, f)**

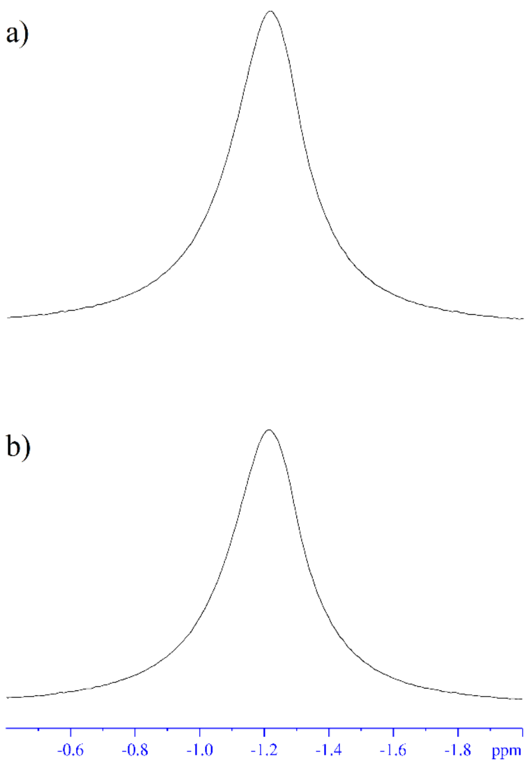

Figure S6. ^{7}Li NMR spectra of the electrolytes **(a)** NPE2 and **(b)** NPE6

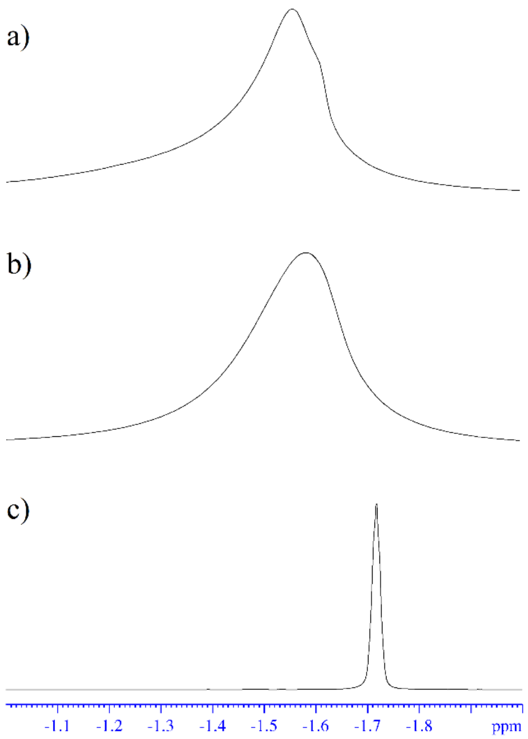

Figure S7. ^{11}B NMR spectra of the electrolytes **(a)** NPE2, **(b)** NPE6 and **(c)** ionic liquid $EMIBF_4$

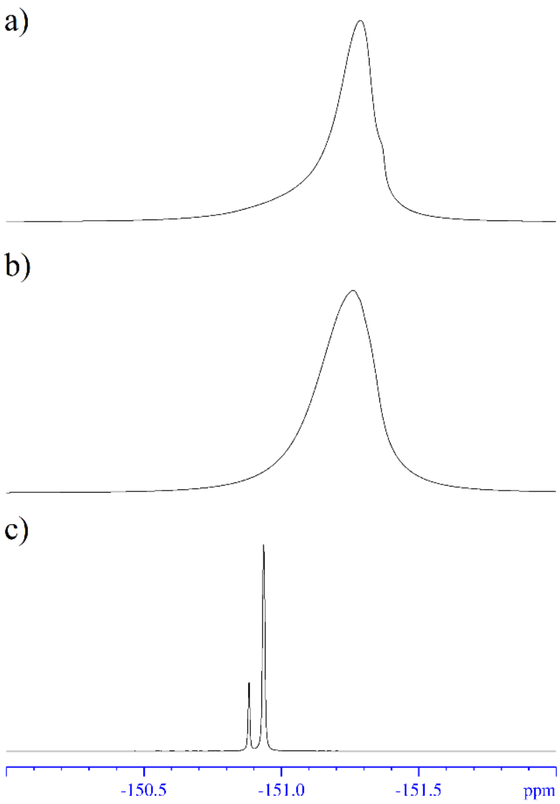

Figure S8.^{19}F NMR spectra of the electrolytes **(a)** NPE2, **(b)** NPE6 and **(c)** ionic liquid EMIBF4

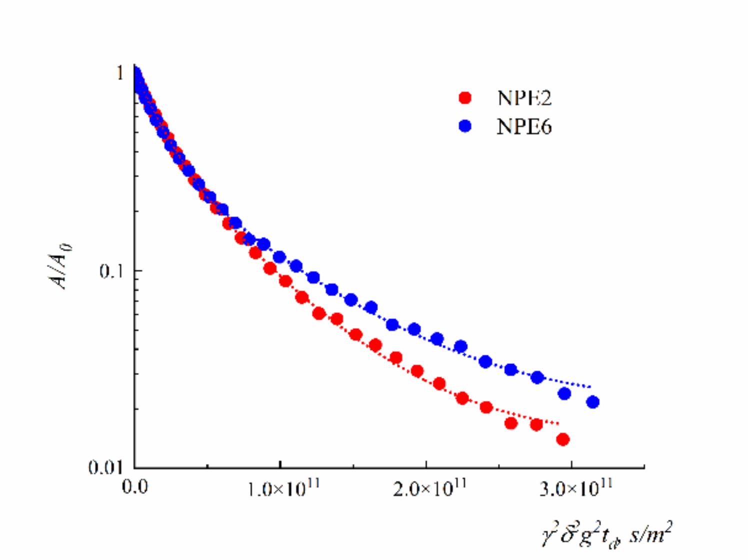

(a)

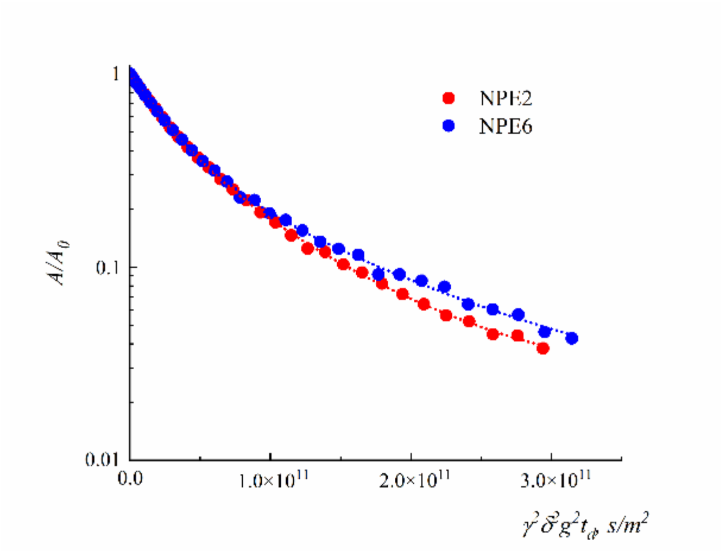

(b)

Figure S9. Diffusion decays of NPE2 and NPE6 on ^{1}H nuclei for **(a)** EC and **(b)** $EMIBF_4$

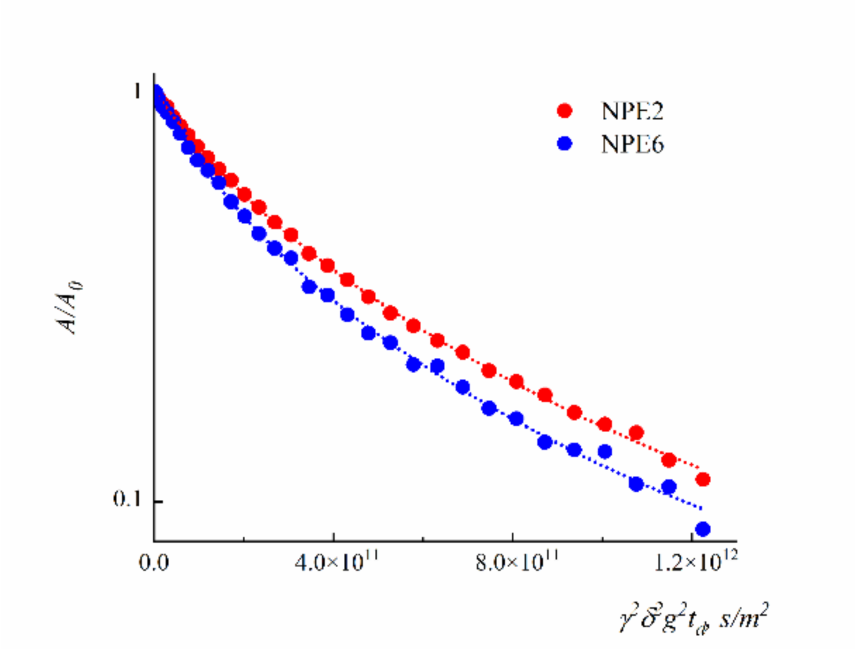

(a)

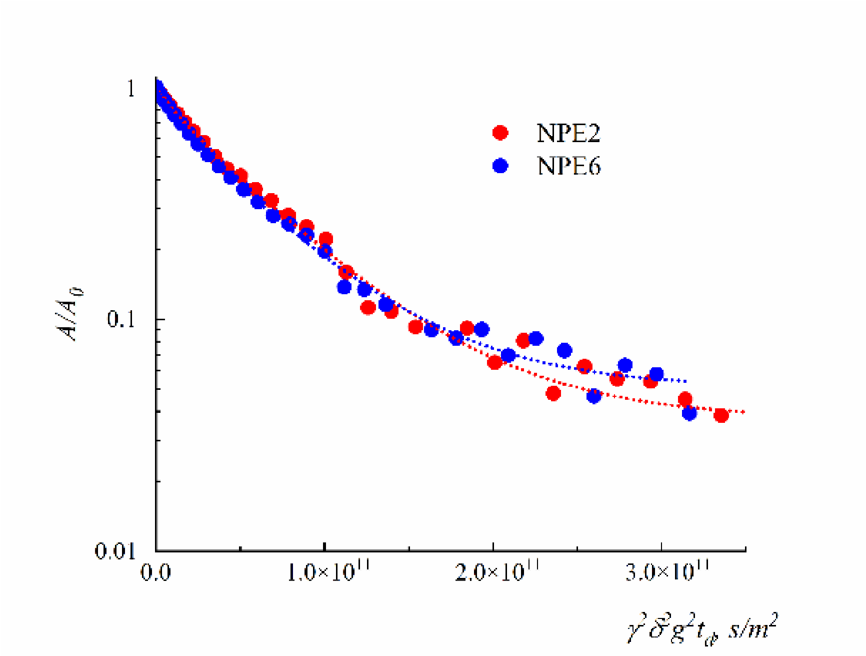

(b)

Figure S10. Diffusion decays of NPE2 and NPE6 on **(a)** ^{7}Li and **(b)** ^{19}F

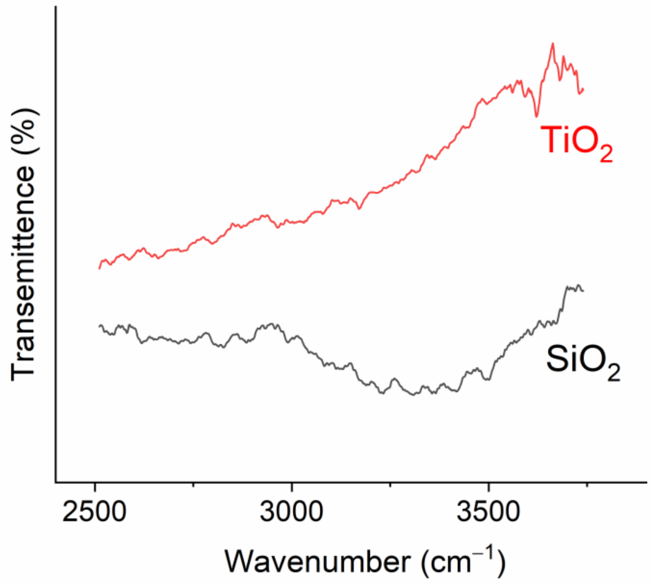

Figure S11. FTIR spectra of the TiO_2 and SiO_2 nanoparticles

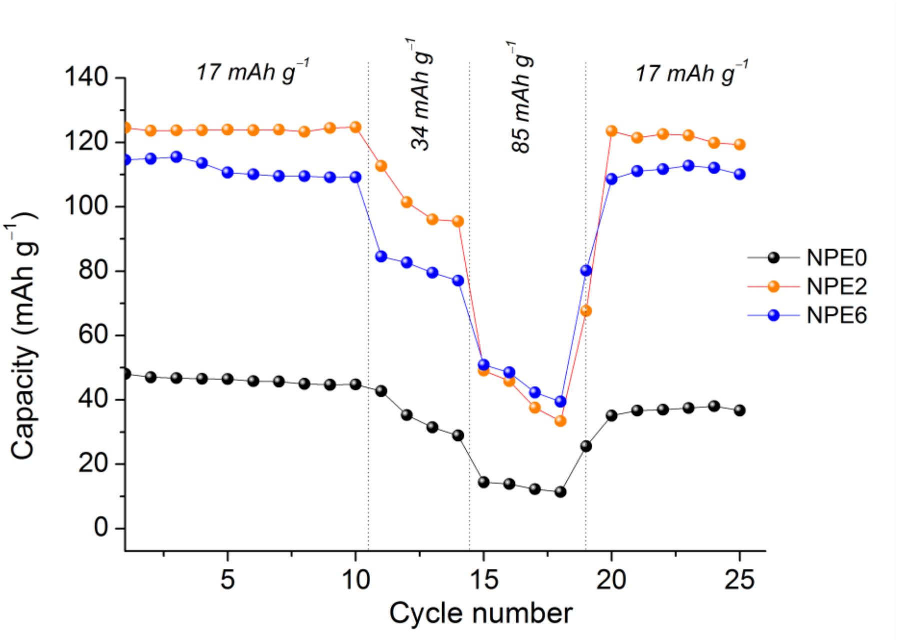

Figure S12. Current rate capability of the Li // $LiFePO_4$ cells in the C/10 to C/2 range.
